# Revolutionising health care: Exploring the latest advances in medical sciences

**DOI:** 10.7189/jogh.13.03042

**Published:** 2023-08-04

**Authors:** Gehendra Mahara, Cuihong Tian, Xiaojia Xu, Wei Wang

**Affiliations:** 1 Clinical Research Center, The First Affiliated Hospital of Shantou University Medical College, Shantou, Guangdong, China; 2 Center for Precision Health, Edith Cowan University, Perth, Australia; 3 Department of Cardiovascular Medicine, The First Affiliated Hospital of Shantou University Medical College, Shantou, Guangdong, China; 4 Shantou University Medical College, Shantou, Guangdong, China; 5 Department of Infection Control, The First Affiliated Hospital of Shantou University Medical College, Shantou, Guangzhou, China

**Figure:**
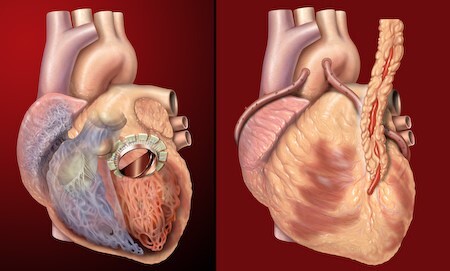
Photo: Human heart, anterior view, artificial valve, coronary bypass. Illustration by Patrick J. Lynch. Source: Flickr, free to use under Creative Commons Attribution 2.5 License (https://creativecommons.org/licenses/by/2.5/).

Recent years have seen a revolution in the domain of medical science, with ground-breaking discoveries changing health care as we once knew it [1]. These advances have considerably improved disease diagnosis, treatment, and management, improving patient outcomes and quality of life [2-5]. These innovations range from the creation of novel medications and treatments to the utilization of cutting-edge technologies. For instance, gene editing technologies like Clustered Regularly Interspaced Palindromic Repeats (CRISPR-Cas9) have opened up new treatment options for genetic illnesses [6], while the development of mRNA vaccines has offered a desperately needed response to the coronavirus disease 2019 (COVID-19) pandemic [7]. Moreover, wearable technology and telemedicine have improved accessibility, convenience, and personalization of health care, whereas 3D printing and nanotechnology breakthroughs have made it possible to create individualized implants and drug delivery systems [8-10]. This article examines some of the most recent developments in medical research and how they might completely change health care delivery.

The selection process for identifying the latest advances in medical sciences for this article was as follows. We aimed to showcase ground-breaking developments with the potential to revolutionise health care practices and significantly impact patient outcomes. We extensively searched reputable scientific journals, conferences, and reports from recognized health care organisations and institutes. We included the novelty and significance of the advancements, their ability to address existing health care challenges, the level of scientific evidence supporting their efficacy, and their potential for widespread adoption and implementation. By utilizing this process, we ensured that the selected advancements represent diverse medical fields and have the capacity to drive significant advancements in patient care, diagnostics, treatment modalities, and health care delivery.

## REGENERATIVE THERAPY TREATMENT

Regenerative medicine is a rapidly growing field that seeks to restore, replace, or regenerate damaged tissues and organs using a variety of approaches, including cell therapy, tissue engineering, and gene therapy [11]. This field has the potential to revolutionise the treatment of many diseases and injuries that are currently incurable or difficult to treat. For example, stem cell therapy has been shown to be effective in treating spinal cord injuries [12], with several studies reporting significant improvements in motor function and sensory perception [13]. Tissue engineering approaches are being developed to replace damaged or diseased organs using 3D printing, such as the liver, pancreas, and heart [11,14]. Gene therapy is being used to target genetic disorders, such as sickle cell anaemia and cystic fibrosis, with promising results [15]. The development of regenerative medicine has the potential to transform the treatment of many diseases and injuries, providing hope for patients with conditions that are currently considered untreatable [16-18].

## DEVELOPMENT OF IMPLANTABLE ARTIFICIAL ORGANS

**Figure 1:**
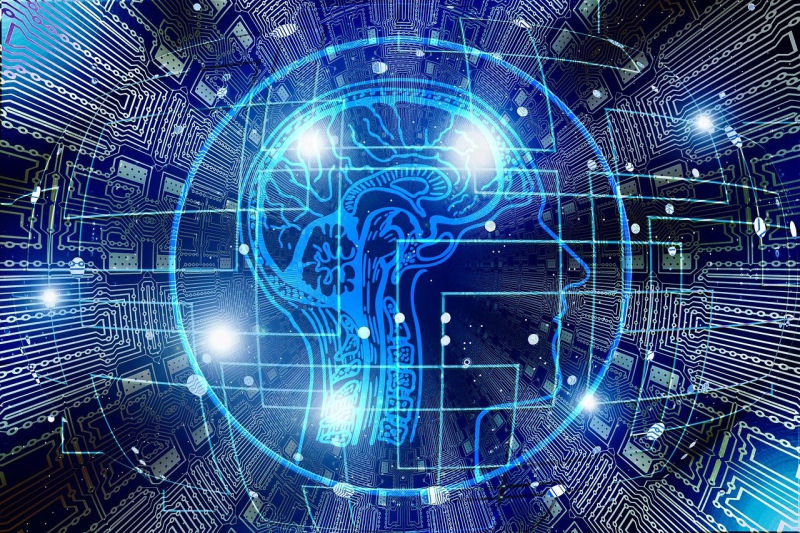
Artificial Intelligence, Brain. Image by Gerd Altmann. Source: Pixabay, free to use under Content License (https://pixabay.com/service/license-summary/).

Various replacement or augmentation devices for organs, such as the eyes, kidneys, heart, muscle, liver, skin, and brain, have been developed due to the creation of implantable artificial organs [4]. Artificial organs can be developed from a number of substances, such as polymers and biological tissues, and are intended to mimic the shape and functionality of actual organs [19]. For instance, the Wearable Artificial Kidney (WAK) has promise for enhancing the quality of life for individuals with end-stage of renal illness [20]. The creation of artificial hearts (**Figure 1**), such as the Total Artificial Heart (TAH), has the potential to extend the lives of patients awaiting heart transplants [21-23].

Furthermore, scientists are developing artificial muscles, liver tissue replicas, skin grafts, and brain implants. For instance, a study by Kolesky et al. [24] reported the successful implantation of a 3D-printed artificial skin graft. Additionally, a study by White [25] and Weng et al. [26] revealed the development of a 3D-printed muscle tissue construct [26]. Although the research into implantable artificial organs is still in its infancy, it has the potential to transform how organ failure is treated and enhance patient outcomes [4].

## ADVANCEMENTS IN NANOTECHNOLOGY IN HEALTH SCIENCE

Another fast-expanding and highly promising area of use for nanotechnology is in the field of medicine. Drugs and other therapeutic substances can be delivered directly to a disease site using nanoparticles because they can target particular cells or tissues in the body [27]. This technology may improve the efficacy of therapies, lessen their negative effects, and potentially enable the treatment of previously incurable diseases [28].

Current developments in nanotechnology have demonstrated considerable promise for the medical field. A study by Foglizzo and Marchio [10] created a multifunctional nano platform that delivered chemotherapeutic medication and an immunomodulatory substance to tumour cells, increasing antitumor activity and minimizing adverse effects. Using nanotechnology, a magnetic resonance imaging (MRI) contrast agent that can specifically target and image pancreatic cancer cells was created [29]. Moreover, nanotechnology has demonstrated promise in the treatment of diseases like brain tumours that were previously incurable. A study by Chen et al. [30] created a nano platform that specifically targeted and delivered medications to brain tumour cells, improving survival rates in a mouse model. These recent developments show how nanotechnology has the potential to enhance therapeutic efficacy, lessen adverse effects, and broaden the scope of diseases that can be treated [31,32].

## DEVELOPMENT OF CRISPR-Cas9 GENE EDITING TECHNOLOGY

A rapidly developing technique called gene editing could revolutionise medicine by enabling researchers to change cells' genetic makeup. CRISPR-Cas9, a promising method for gene editing, allows for accurate targeting and editing of particular regions of the genome [33]. Genetic disorders like cystic fibrosis and sickle cell anaemia, which were once thought to be incurable, could potentially be cured because of this technique [34,35]. Also, scientists are looking at its therapeutic potential for a number of illnesses, such as Alzheimer’s disease, human immunodeficiency virus (HIV), and cancer [34,36].

Yet there are also moral questions raised by using gene editing on people, so it's important to use the technology sensibly and morally. Until the hazards and moral issues surrounding germline editing, which edits the genes that can be passed on to future generations, are better known, a group of scientists called for a moratorium on its clinical usage in 2019 [37].

## ARTIFICIAL INTELLIGENCE (AI) FOR MEDICAL SCIENCE

Recent years have seen considerable advancements in the use of artificial intelligence (AI) and machine learning in the health care industry. In order to find trends and forecast health outcomes, AI systems can evaluate enormous amounts of medical data, including images, test results, and patient records [38]. This may result in more accurate diagnosis, individualized treatment strategies, and effective patient monitoring.

The promise of AI in health care has been proved by a number of studies. For instance, Esteva et al. [39], created an AI model with skin cancer detection accuracy on par with dermatologists. Rajkomar et al. [40] use of machine learning to forecast patient mortality and hospital readmission rates may aid health care professionals in identifying patients who need more care. Moreover, Chung et al. [41], created an AI algorithm that could anticipate the onset of psychosis in individuals who had clinical high-risk signs.

Predicting the risk of cardiovascular illness using AI has also shown promise. For example, Khera et al. [42] developed a model using machine learning to identify patients with a high risk of developing heart disease, potentially allowing for early intervention and preventative measures.

Yet, there are also issues with using AI in health care that need to be resolved, such as the requirement for strong data protection and ethical concerns with the use of AI algorithms to clinical decision-making [43].

## CHIMERIC ANTIGEN RECEPTOR (CAR) T-CELL THERAPY TO TREAT CANCER

Chimeric Antigen Receptor (CAR) T-cell therapy, a form of immunotherapy that employs T cells to recognize and target cancer cells, depends heavily on genetically transformed T cells [44]. Recent studies have demonstrated that CAR T treatment is very effective in treating a range of lymphoma types, including diffuse large B-cell lymphoma and mantle cell lymphoma [45,46].

Despite the positive outcomes, CAR T therapy has drawbacks, such as a high price and risk for toxicity. In order to increase the effectiveness and safety of CAR T treatment and broaden its use to treat additional cancer types, research is now being done by Ren et al. [47]. For instance, a recent study by Yang et al. [48] discovered that multiple myeloma, a kind of blood cancer, that has relapsed or become resistant to treatment, can be effectively treated with CAR T therapy that targets the B-cell maturation antigen (BCMA). Researchers are also investigating combination therapies, which couple CAR T therapy with additional medications, including checkpoint inhibitors, to enhance results [49].

## DEVELOPMENT OF mRNA VACCINE

The development of mRNA vaccines has been a significant milestone in the fight against COVID-19 [50]. The Pfizer-BioNTech and Moderna mRNA vaccines have demonstrated remarkable efficacy and safety profiles in preventing COVID-19 infection and its complications [7,51,52]. The mRNA technology used in these vaccines has several advantages over traditional vaccine production methods, including faster development and manufacturing times, lower production costs, and greater flexibility in responding to emerging viral variants [53,54].

Clinical trials of the Pfizer-BioNTech and Moderna vaccines have shown high levels of protection against COVID-19. A study by Polack et al. [55] found that the Pfizer-BioNTech vaccine had an efficacy rate of 95% in preventing COVID-19 infection, while a study by Baden et al. [56] reported a similar efficacy rate of 94.1% for the Moderna vaccine. Additionally, real-world data has confirmed the high effectiveness of mRNA vaccines in preventing severe disease, hospitalization, and death caused by COVID-19 [57].

Another company that has been working on developing mRNA vaccines for COVID-19 is Novavax [58]. The company's vaccine candidate combines mRNA technology with nanoparticles to enhance the body's immune response [59]. In clinical trials, the vaccine demonstrated efficacy against both the original strain of COVID-19 and certain variants of the virus [60].

Companies such as Moderna and BioNTech are now exploring the potential of mRNA vaccines for a wide range of illnesses, including cancer and influenza [61]. The development of mRNA vaccines also holds promise for creating rapid responses to new and emerging infectious diseases, as the technology allows for quick adaptation to new viral strains [7,54,61,62].

Overall, the development of mRNA vaccines for COVID-19 represents a significant breakthrough in vaccine technology, with potential implications for future disease prevention and treatment [53].

## ADVANCES IN 3D PRINTING FOR MEDICAL APPLICATIONS

The development of complex anatomical models, prostheses, implants, and drug delivery systems has been made possible by advances in 3D printing technology [8]. 3D printing has enabled the development of custom-made implants, reducing the need for invasive surgeries and improving patient outcomes. The successful implantation of 3D printed titanium-mesh implants for the repair of bone deformities was described in a study by Ma et al. [63]. Anatomical models that have been 3D printed have been proven to be useful for planning surgeries and advancing medical knowledge. The use of 3D printed models for surgical planning in complicated craniofacial patients was reported in a study by Charbe et al. [64]. The development of 3D printing technology has the potential to revolutionise the medical industry by enabling more individualized and efficient patient care [65].

## TELEMEDICINE TO PROVIDE REMOTE CARE

Over the past few years, telemedicine – the use of technology to deliver medical treatments remotely – has grown in popularity, especially during the COVID-19 pandemic [66]. Telemedicine allows health care providers to offer virtual consultations, monitor patients remotely, and provide access to medical services in areas with limited health care resources [67]. Telemedicine was linked to better health care access and outcomes for patients with cardiovascular disease during the COVID-19 pandemic [9]. Telemedicine also has the potential to lower medical expenses and raise patient satisfaction. High levels of patient satisfaction with teleconsultations for dermatology services were observed in a study by Nicholson et al. [68]. Telemedicine use is anticipated to increase over the next few years, which might have a significant impact on how health care is delivered in the future [9,69].

## VERTUAL REALITY IN MEDICAL TRAINING

Medical students can practice and hone their skills in a safe and controlled environment with the help of virtual reality (VR), which has grown in popularity in recent years [70]. Students can practice medical procedures and scenarios using VR technology, which helps them become more adept at diagnosing and treating patients [71]. According to a recent study by Yiasemidou et al. [72], medical students' performance and confidence improved when VR was used for surgical instruction. Moreover, using VR technology can replace animal or cadaveric models in training for less common medical operations. The effective use of VR technology in training for transesophageal echocardiography was described in a study by Arango et al. [73]. The use of VR in medical education has the potential to raise the standard of medical instruction and increase patient safety [74].

## DEVELOPMENT OF WEARABLE DEVICES FOR HEALTH MONITORING

The development of wearable health monitoring technology has completely revolutionised how people track and manage their health [75]. Individuals can receive real-time feedback on their health state by using wearable devices, such as fitness trackers and smartwatches, which can gather data on physical activity, heart rate, blood oxygen saturation, sleep habits, and other health markers [76]. These devices capture data that can be analysed to find trends and patterns that can provide important information about a person's general health and well-being [77,78]. According to research by Patel et al. [79], adult users of wearable technology had increases in physical activity and weight loss. Moreover, wearable technology can be used to monitor patients with chronic illnesses remotely, enabling health care professionals to monitor patient progress and take appropriate action as needed. According to a study by Gautam et al. [80], wearable devices are useful for remotely monitoring patients with heart failure [80,81]. By encouraging early disease identification and prevention, wearable health monitoring technology has the potential to enhance health outcomes and save health care costs [78].

## CONCLUSIONS

In conclusion, the most recent developments in medical science have the potential to completely revolutionise the way health care is provided and greatly enhance patient outcomes. With the advent of modern technologies like telemedicine, gene editing, and AI, doctors are now able to detect and treat illnesses more precisely and effectively. Moreover, the application of nanotechnology, 3D printing, and regenerative medicine is bringing about ground-breaking treatments for previously incurable diseases. The advances being made in medical science are genuinely astonishing and give hope for a healthier future, even though there are still obstacles to be addressed. In the years to come, we may anticipate even more interesting advances with ongoing innovation and investment.
